# High levels of *CRBN* isoform lacking IMiDs binding domain predicts for a worse response to IMiDs-based upfront therapy in newly diagnosed myeloma patients

**DOI:** 10.1007/s10238-023-01205-y

**Published:** 2023-10-10

**Authors:** Enrica Borsi, Gaia Mazzocchetti, Angela Flores Dico, Ilaria Vigliotta, Marina Martello, Andrea Poletti, Vincenza Solli, Silvia Armuzzi, Barbara Taurisano, Ajsi Kanapari, Ignazia Pistis, Elena Zamagni, Paola Tacchetti, Lucia Pantani, Katia Mancuso, Serena Rocchi, Ilaria Rizzello, Michele Cavo, Carolina Terragna

**Affiliations:** 1 IRCCS Azienda Ospedaliero-Universitaria di Bologna–Istituto di Ematologia “Seràgnoli”, Bologna, Italy; 2 DIMEC–Dipartimento di Scienze Mediche e Chirurgiche, Università di Bologna, Bologna, Italy; 3 Labgen s.n.c., Montallegro, Italy

**Keywords:** Multiple myeloma, IMiDs, *CRBN* isoforms, *CRBN* exon10 spliced, IMiDs resistance

## Abstract

In recent years, the immunoderivative (IMiD) agents have been extensively used for the treatment of multiple myeloma (MM). IMiDs and their newer derivatives CRBN E3 ligase modulator bind the E3 ligase substrate recognition adapter protein cereblon (CRBN), which has been recognized as one of the IMiDs’ direct target proteins, and it is essential for the therapeutic effect of these agents.

High expression of CRBN was associated with improved clinical response in patients with MM treated with IMiDs, further confirming that the expression of IMiDs’ direct target protein CRBN is required for the anti-MM activity. CRBN’s central role as a target of IMiDs suggests potential utility as a predictive biomarker of response or resistance to IMiDs therapy. Additionally, the presence of alternatively spliced variants of CRBN in MM cells, especially those lacking the drug-binding domain for IMiDs, raise questions concerning their potential biological function, making difficult the transcript measurement, which leads to inaccurate overestimation of full-length CRBN transcripts. In sight of this, in the present study, we evaluated the CRBN expression, both full-length and spliced isoforms, by using real-time assay data from 87 patients and RNA sequencing data from 50 patients (*n* = 137 newly diagnosed MM patients), aiming at defining CRBN’s role as a predictive biomarker for response to IMiDs-based induction therapy. We found that the expression level of the spliced isoform tends to be higher in not-responding patients, confirming that the presence of a more CRBN spliced transcript predicts for lack of IMiDs response.

**Supplementary Information:**

The online version contains supplementary material available at 10.1007/s10238-023-01205-y.

## Introduction

The use of immunomodulatory drugs (IMiDs) have brought many advantages in the treatment of hematological disease particularly in patients with multiple myeloma (MM) [1]. Despite the teratogenic effect [2], the IMiDs’ molecular mechanism of action proves an important therapeutic outcome on MM, since IMiDs both interfere with the growth and the development of neoplastic cells and stimulate the bone marrow environment to produce factors with anti-angiogenic, pro-apoptotic, anti-proliferative, and anti-inflammatory properties.

A major breakthrough in deciphering the molecular mechanism of IMiDs came using zebrafish and chicken embryo modeling, with the discovery that thalidomide bounds directly to cereblon (CRBN) and that this interaction was necessary for its teratogenic effects [2]. Soon after, it was shown that CRBN expression was required for the antimyeloma activity of IMiDs [3]. CRBN is part of a multi-proteic complex called CRL4^CRBN^, an E3 ubiquitin ligase, which includes damage DNA binding protein 1 (DDB1), Cullin 4 (Cul-4), and regulator of Cullin1 (ROC1) [4, 5]. In physiological conditions, this complex exerts both an auto-ubiquitination activity as well as the ubiquitination of intracellular substrates, which direct them toward a proteasome-mediated degradation [6]. Targeting CRBN by IMiDs modifies its substrate specificity toward non-physiological proteins which are subsequently ubiquitinated and degraded by the proteasome [5, 7–10]. Specifically, in the presence of IMiDs, the complex becomes unable to auto-ubiquitinate and redirects its ubiquitination activity toward novel targets, among which are Ikaros and Aiolos (IKZF1 and IKZF3, respectively), two transcription factors mainly involved in the lymphopoiesis [11, 12]. One of the downstream effects of IKZF1 and IKZF3 degradation is the inhibition of IRF-4, which finally results cytotoxic for MM cells [13]. CRBN, acting as the CRL4^CRBN^ complex’ receptor, is essential for the anti-tumoral activity of the IMiDs [3]. Moreover, IMiD drug-induced degradation of Ikaros and Aiolos also occurs in T lymphocytes and is involved in T-cell co-stimulation via down-regulation of interleukin-2 transcription [10]. CSNK1A1 (CK1α) was also identified as a lenalidomide-specific neosubstrate with clinical relevance to del(5*q*) myelodysplastic syndrome [14, 15]. Additionally, GSPT1 is a CC-885-specific neosubstrate that mediates the broad-spectrum anticancer proprieties of CC-885 [16]. Notably, genomic defects in CRBN, including mutations, copy number loss, transcriptomic aberrations (epigenetic, RNA splicing/stability), and specific exon10-deleted splice transcript variant, increase in IMiD-resistant relapsed and refractory MM (RRMM) patients. Indeed, almost one-third of MM patients have genetic alterations in CRBN by the time they are refractory to IMiDs (both lenalidomide and pomalidomide), and these genetic changes are associated with inferior outcomes [2, 17–21]. The CRBN gene contains 11 exons encoding a protein comprising 442 amino acids residues with its C-terminal portion (encoded in part by exon10) containing the drug-biding domain. *CRBN* spliced variant has been associated with lenalidomide refractoriness, and unlike mutation or copy loss, *CRBN* exon10 splice isoform was observed in newly diagnosed MM (NDMM) patients at ~ 2.9% prevalence which increased to almost 30% in IMiD-resistant RRMM and was a prognostic marker for poor outcome in both disease setting [18]. Disruption of CRBN activity is the best understood mechanism of IMiD resistance in MM. As CRBN is necessary for IMiD activity [3], either quantitative or qualitative CRBN defects may contribute to IMiD ineffectiveness or resistance. It has been shown that the efficacy of a thalidomide analog may be influenced by both the level of CRBN expression and the levels of alternative substrates [7, 22, 23]. Substrate competition is a novel mediator of resistance to this class of drugs, whereby increased expression of a secondary protein drug target unrelated to canonical drug activity can lead to resistance [24]. Indeed, ARID2 it has been proposed as a pomalidomide-dependent CRL4^CRBN^ substrate in MM cells, and its degradation is necessary for the anti-MM activity of pomalidomide. Several lines of evidence indicated that pomalidomide-induced ARID2 degradation is mediated by BRD7, suggesting a new mechanism of substrate recognition by CRL4^CRBN^ [25]. The authors shown that pomalidomide can induce formation of the CRBN–BRD7–ARID2 trimeric complex even if CRBN–BRD7 and BRD7–ARID2 interactions are essentially constitutive. BRD7 exists either in a free form or an ARID2-bound form at an unknown ratio. In the absence of pomalidomide, the two forms bind equally to CRBN: in the presence of pomalidomide, however, the ARID2-bound form preferentially binds to CRBN [25]. Aberrations to any or all of these factors could cause IMiD resistance.

Nevertheless, the biological basis for the prognostic and/or predictive role of CRBN (e.g., as a biomarker of resistance to IMiDs) is still unknown.

Here, we analyzed a cohort of NDMM patients who received an IMiDs induction therapy and we evaluated the total *CRBN* expression level at baseline, to establish its role, already at diagnosis, as biomarker to predict the response to IMiDs therapy. Particular attention has been paid to the presence of different *CRBN* isoforms, especially those missing the IMiDs binding domain (i.e., *CRBN* lacking the exon 10), which might alter the quantification results, but also allegedly impact on patients’ outcome. Gene expression profiles of patients, either responding to IMiDs or not based therapy, were also determined to identify putative pathways involved in IMiDs resistance.

## Materials and methods

### Patients

An overall number of 137 of diagnostic samples from newly diagnosed MM (NDMM) patients, belonging to two different datasets, were enrolled in this study. Briefly, Bologna cohort (MM-BO) includes a total of 87 NDMM, homogeneously treated with IMiDs-based induction therapy (Thalidomide–Dexamethasone (TD, *n* = 86) or Bortezomib plus TD (VTD, *n* = 1) regimens), and then exposed to consolidation therapy after autologous peripheral blood stem cell transplantation (ASCT). The remaining patients (*n* = 50) were enrolled in Multiple Myeloma Research Foundation (MMRF) CoMMpass trial [26], and samples were selected based on IMiDs first-line therapy (Elotuzumab–Lenalidomide–Dexamethasone, *n* = 2, Lenalidomide, *n* = 4, Lenalidomide–BIAXIN–Dexamethasone, *n* = 1, Lenalidomide–Dexamethasone, *n* = 40, Lenalidomide–Methylprednisolone, *n* = 1, Lenalidomide–Prednisone, *n* = 1, Thalidomide–Melphalan–Prednisone, *n* = 1). Three patients treated with Lenalidomide–Dexamethasone induction therapy were then exposed to ASCT. The MMRF CoMMpass study is a prospective observational clinical trial (NCT01454297) with comprehensive genomic and transcriptomic characterization of NDMM patients, funded and managed by the MMRF. The study is ongoing, with data released regularly for research use via the MMRF research gateway, https://research.themmrf.org. In this study, we used Interim Analysis (IA) 18. Response to therapy was evaluated according to the International Myeloma Working Group criteria (IMWG) [27]. Patients’ baseline clinical and cytogenetic characteristics are summarized in Table 1.

**Table 1:** Patients’ clinical characteristics at baseline

Characteristic	MM-BO (*n* = 87)	CoMMpass (*n* = 50)	Total (*n* = 137)
Median age, y (range)	55 (23–66)	71 (51–90)	61 (23–90)
*Sex, n (%)*			
Male	53 (60)	28 (56)	81 (59)
Female	34 (40)	22 (44)	56 (41)
*ISS, n (%)*			
I	24 (28)	22 (44)	39 (28.5)
II	34 (39)	11 (22)	46 (33.5)
III	15 (17)	15 (30)	23 (16.8)
na	14 (16)	2 (4)	29 (21.2)
*R-ISS, n (%)*			
I	0	18 (36)	18 (13.3)
II	44 (51)	28 (56)	72 (52.5)
III	3 (3)	2 (4)	5 (3.6)
na	40 (46)	2 (4)	42 (30.6)
*Cytogenetic abnormalities, n (%)*			
Del_17p	4 (5,3)^*na for 12 pts^	9 (18)	13 (10.4)^*na for 12 pts^
Del_13	36 (42,86)^*na for 3 pts^	7 (14)	43 (32)^*na for 3 pts^
Del_1p	na	13 (26)	13 (26)^*na for 87 pts^
Amp_1q	na	5 (10)	5 (10)^*na for 87 pts^
*Translocations, n (%)*			
Translocated	20 (23)	13 (26)	33 (24)
Non-translocated	–	37 (74)	37 (27)
na	67 (77)	–	67 (49)
*Hemoglobin* (g/L)			
Median (IQR)	11 (3–15.6)	6.231 (7.05–9.982)	9.98 (11.4–15.6)
Mean (SD)	11.14 (1.71)	6.40 (1.14)	9.41 (2.75)
*Platelets* (× 10^9^ per L)			
Median (IQR)	230 (271.5–569)	205.5 (267.5–439)	226 (271–569)
Mean (SD)	238.14 (85.52)	223.56 (79.42)	232.82 (83.35)
*b2-microglobulin* (mg/L)			
Median (IQR)	3.45 (4.89–23.8)	4.16 (5,80–17.5)	3,50 (5.17–23.8)
Mean (SD)	4.22 (3.11)	4.82 (3.16)^*na for2 pts^	4.43 (3,13)^*na for 3 pts^
*Albumin* (g/dL)			
Median (IQR)	3.9 (4.18–4.9)	3.55 (3.80–4.4)	3.70 (4–4.9)
Mean (SD)	3.83 (0.54)^*na for 27 pts^	3.41 (0.53)^*na for 2 pts^	3.64 (0.54)^*na for 29 pts^
*Creatinine* (mmol/L)			
Median (IQR)	1 (1.25–14.5)	0.97 (1.23–1.85)	1(1.24–14.5)
Mean (SD)	1.72 (1.94)^*na for 2 pts^	1.04 (0.36)^*na for 1 pts^	1.31 (1.57)^*na for 3 pts^

*Y* years, *n* number, *ISS* international staging system, *R-ISS* revised ISS, *na not available, *IQR* interquartile range, *SD* standard deviation, *pts* patients

### Cells isolation

Mononuclear cells from diagnostic bone marrow (BM) aspirates were isolated by density gradient centrifugation over Ficoll-Paque Plus (Amersham Biosciences, Piscataway, NJ, USA). BM-CD138^+^ cells isolation and purity evaluation were performed as previously published [28–30]. Briefly, the purity of MM cells was confirmed by flow cytometry, and only cases enriched for more than 85% were included in the study. Patients’ samples were collected after informed consent.

### RNA isolation and quantitative real-time PCR (qPCR)

Total RNA was isolated using RNeasy total RNA isolation kit (QIAGEN, Valencia, CA) with an automated RNA extraction method according to the manufacturer’s instructions (QIAcube, QIAGEN, Valencia, CA). 100 ng of total RNA was reverse transcribed using SuperScript™ III First-Strand Synthesis System and random hexamers (Inivtrogen Life Technologies). All reactions were performed in triplicate using the LightCycler 480 Instrument (Roche, Applied science) in a total volume of 20 μl. An absolute quantification was performed using probe assays (PrimeTime® qPCR 5′ Nuclease Assay, IDT) both for *CRBN* full-length (FL), using a standard pre-designed assay with the probe localized between exons 9 and 11, and for *CRBN* isoform, lacking exons 8 and 10 (henceforth called *CRBN* exon10-spliced), with the probe localized between exons junction 7–9. GAPDH was used as endogenous control to normalize the level expression of *CRBN*. *CRBN* available for IMiDs binding was expressed for each patient as ratio between *CRBN* exon10-spliced and *CRBN*-FL isoforms. Analysis of relative transcript levels was calculated using the delta-delta Ct method (ΔΔCT). For CoMMpass dataset, *CRBN* expression analysis was performed by using RNA sequencing (RNAseq) online data of diagnostic samples.

### CoMMpass gene expression data analysis

Together with MM-BO results, RNAseq expression data about the two *CRBN* isoforms from CoMMpass project were integrated. Specifically, we selected the transcript-level counts of the isoform ENST00000231948 (*CRBN*-FL) and ENST00000424814 (*CRBN* exon10-spliced) from baseline CD138 + samples of 50 patients having first-line IMID-based therapy. Instead, the gene-level count matrices were used to perform a differential expression gene (DEG) analysis with the R package DEseq2 [31], pre-filtering only genes that have at least 5 reads total. Following, to study the pathways of the biological processes that were mainly expressed or not in the compared patient groups, a gene set enrichment analysis (GSEA) was done with R package clusterProfiler [32].

### Clinical and statistical analysis

Ratios *CRBN* exon10-spliced/*CRBN*-FL (mRNA) were correlated to response to therapy after front-line IMiDs-based induction treatment. *CRBN* ratios were calculated based on the *CRBN* gene expression data derived from qPCR (MM-BO dataset) and RNA sequencing (RNAseq for CoMMpass samples) data. Patients were clustered according to their response to induction therapy (first response), and patients achieving at least Very Good Partial Response (VGPR) were compared to patients failing this objective. Ratios of the two populations were compared through Wilcoxon rank sum test. Cox regression model was used to assess the relationship between risk factors and patient’s survival times. Survival curves of each model were visualized with the Kaplan–Meier method and compared with long-rank test. Fisher’s test and generalized linear model (GLM) were chosen for univariate and multivariate analyses, respectively. All statistical analyses were performed with R software with *survival, survminer, gmodels*, and *stats* packages.

## Results

### Baseline *CRBN* isoforms expression level in newly diagnosed MM patients

We first determined the baseline expression of *CRBN* isoforms by analyzing quantitative real-time PCR (qPCR) data from 87 and RNA sequencing (RNASeq) data from 50 NDMM patients (MM-BO and CoMMpass cohort, respectively) who received an IMiD-based induction therapy (summary patients characteristics are listed in Supplementary Table 1). A comparison of the patients’ baseline characteristics revealed no significant differences between the IMiD-based treatment groups, except for higher rates of male/female patients (60% vs. 40% and 56% vs. 44% in MM-BO and CoMMpass dataset, respectively) (Table 1).

**Fig. 1:**
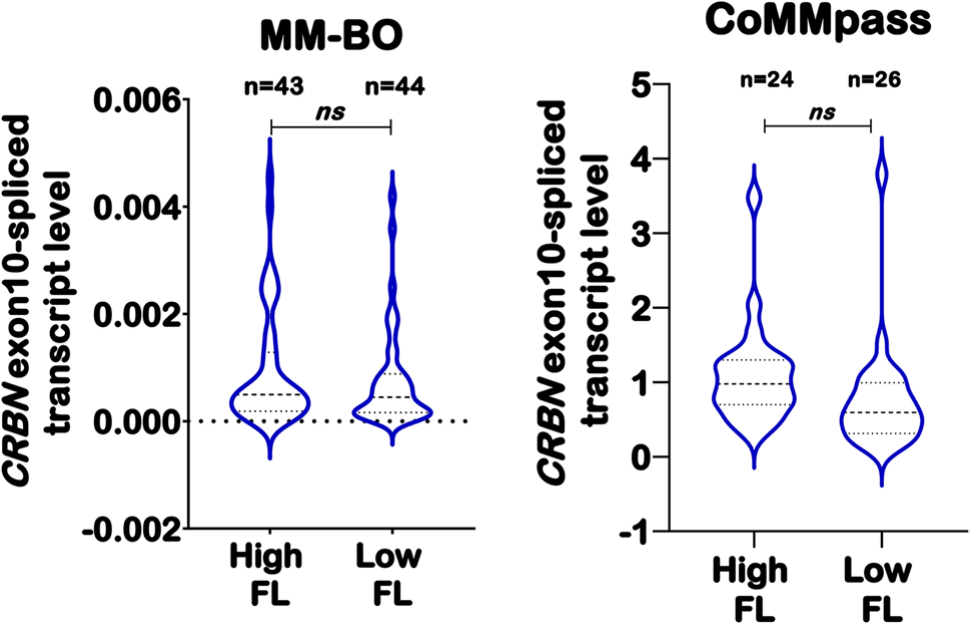
Baseline expression levels of *CRBN* isoforms in newly diagnosed MM patients. Violin plots show the level of *CRBN*-FL and *CRBN* exon10-spliced variants in MM-BO (*n* = 87) and CoMMpass (*n* = 50) datasets. *CRBN* isoforms are expressed as absolute mRNA transcript level. Overall, *CRBN* exon10-spliced variant does not change with respect to the level of full-length isoform in the two subgroups. High FL = high *CRBN*-FL, low FL = low *CRBN*-FL

Overall, *CRBN* expression levels of both *CRBN*-FL and *CRBN* exon10-spliced isoforms were lower in our dataset compared to CoMMpass subgroup (Supplementary Table 1), differences mainly ascribed to the methods used for measuring *CRBN* transcript. In particular, the analysis of the *CRBN* exon10-spliced isoform mRNA level in the two datasets highlighted a wide range of deleted isoform expression values (for MM-BO dataset range 0.0000260–0.004549, and for CoMMpass dataset range 0.104776–3.790971, respectively). We then determined the optimum sample cut-off for both *CRBN* isoforms by using the medians value in each dataset to establish the high and low-expression range (MM-BO: *CRBN* exon10-spliced 0.000494 and *CRBN*-FL 0.021798, respectively; CoMMpass: *CRBN* exon10-spliced 0.774215 and *CRBN*-FL 4.944441, respectively). According to the established cut-offs, we compared the distribution of *CRBN* isoforms between samples in each dataset, and we found that *CRBN* exon10-spliced expression did not significantly vary between cases with high *vs.* low *CRBN*-FL isoform in the two cohorts (Fig. 1).

### The ratio between* CRBN* exon10-spliced and *CRBN*-FL transcripts correlate with response to therapy

According to the response rate evaluated referring to the IMWG criteria [27], all patients (*n* = 137) were stratified in “responders” (R) (i.e., achieving at least a Very Good Partial Response, VGPR) and “non-responders” (NR) (i.e., achieving a suboptimal response, < VGPR).

**Fig. 2:**
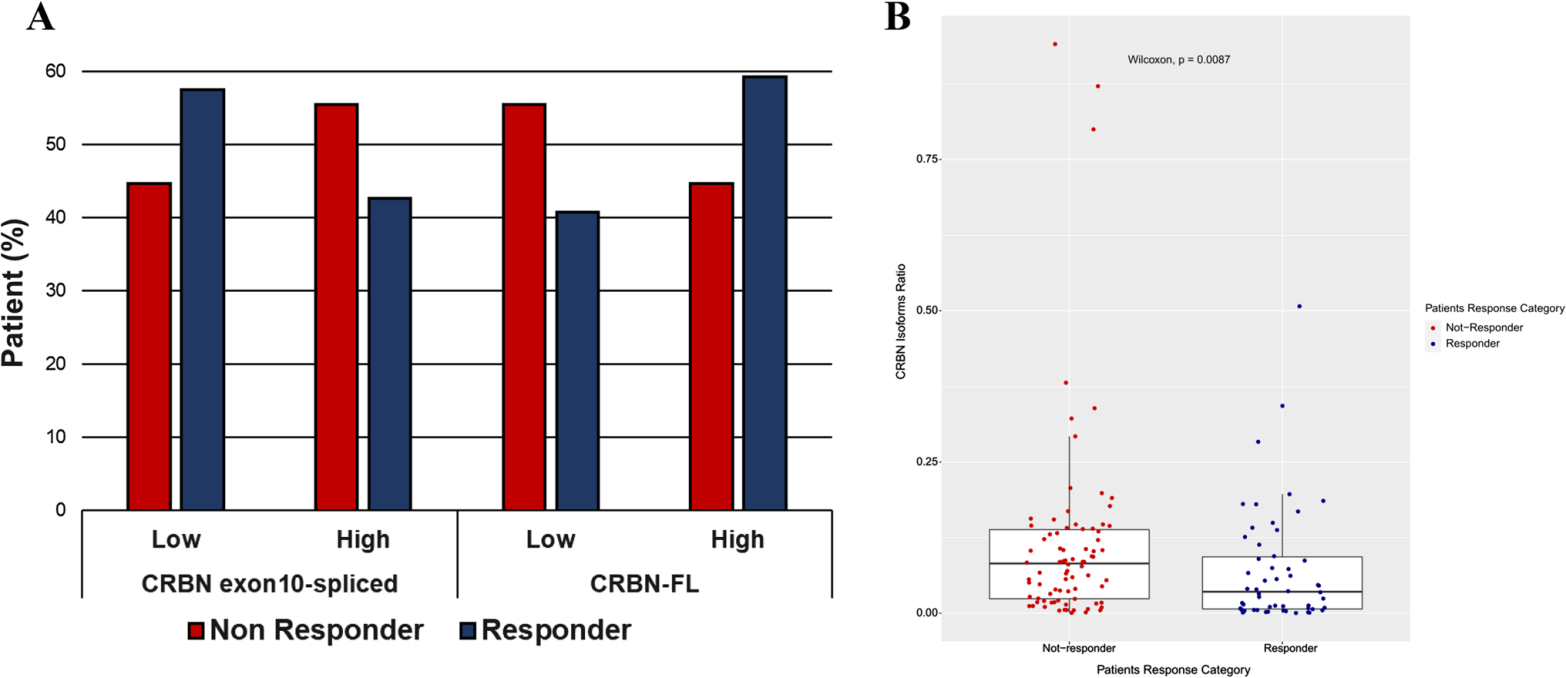
Distribution of *CRBN* isoforms and correlation with response to IMiDs first-line induction therapy. **A** distribution of *CRBN*-FL and *CRBN* exon10-spliced in MM patients stratified, according to the response rate evaluated referring to the IMWG criteria^13^ in responder and non-responder. The definition of high- or low-expression *CRBN* mRNA level of both variants was defined based on the median value calculated on all MM samples (*n* = 137). **B** Change in ratio of *CRBN* exon10-spliced/*CRBN*-FL transcripts between non-responder and responder patients. Significant differences detected by Wilcoxon signed-rank test (nonparametric)

Overall, 54 out of 137 patients (i.e., 39.42%) were defined as R (10 achieved Complete Response, CR, 13 achieved near Complete Response, nCR, and 31 achieved VGPR). The remaining 83 patients (i.e., 60.58%) were included in the NR group (27 in Stable Disease, *SD*, 48 achieved Partial Response, PR, and 8 achieved Progressive Disease, PD). Most of the patients received an IMiDs-based induction therapy (*n* = 115, with Lenalidomide *n* = 29 or Thalidomide *n* = 86), whereas the remaining patients received a combined Bortezomib-IMiDs-based first-line therapy (*n* = 22) (patients characteristic summarized in Supplementary Table 1). We compared the *CRBN* isoforms expression level between subgroups (R *vs*. NR), and we found that 57% (31 out of 54) of R patients showed lower expression of *CRBN* exon10-spliced isoform compared to NR (45%, 37 out of 83), whereas 59% (32 out of 54) showed a higher *CRBN*-FL isoform level respect to NR patients (45%, 37 out of 83) (Fig. 2A). Conversely, NR patients showed lower *CRBN*-FL isoform level (55%, 46 out of 83) compared to R patients (41%, 22 out of 54). Although we did not observe significant differences in terms of transcript level between the two patients’ subgroups (Fisher’s exact test, Low *vs* High *CRBN* exon10-spliced *p* = 0.1640 and Low *vs* High *CRBN*-FL *p* = 0.1161), these results suggested that a higher percentage of deleted *CRBN* predicted for lack of IMiDs response, even though some overlaps still persist between the expression levels of the deleted *CRBN* isoform among the two subgroups of patients.

**Fig. 3:**
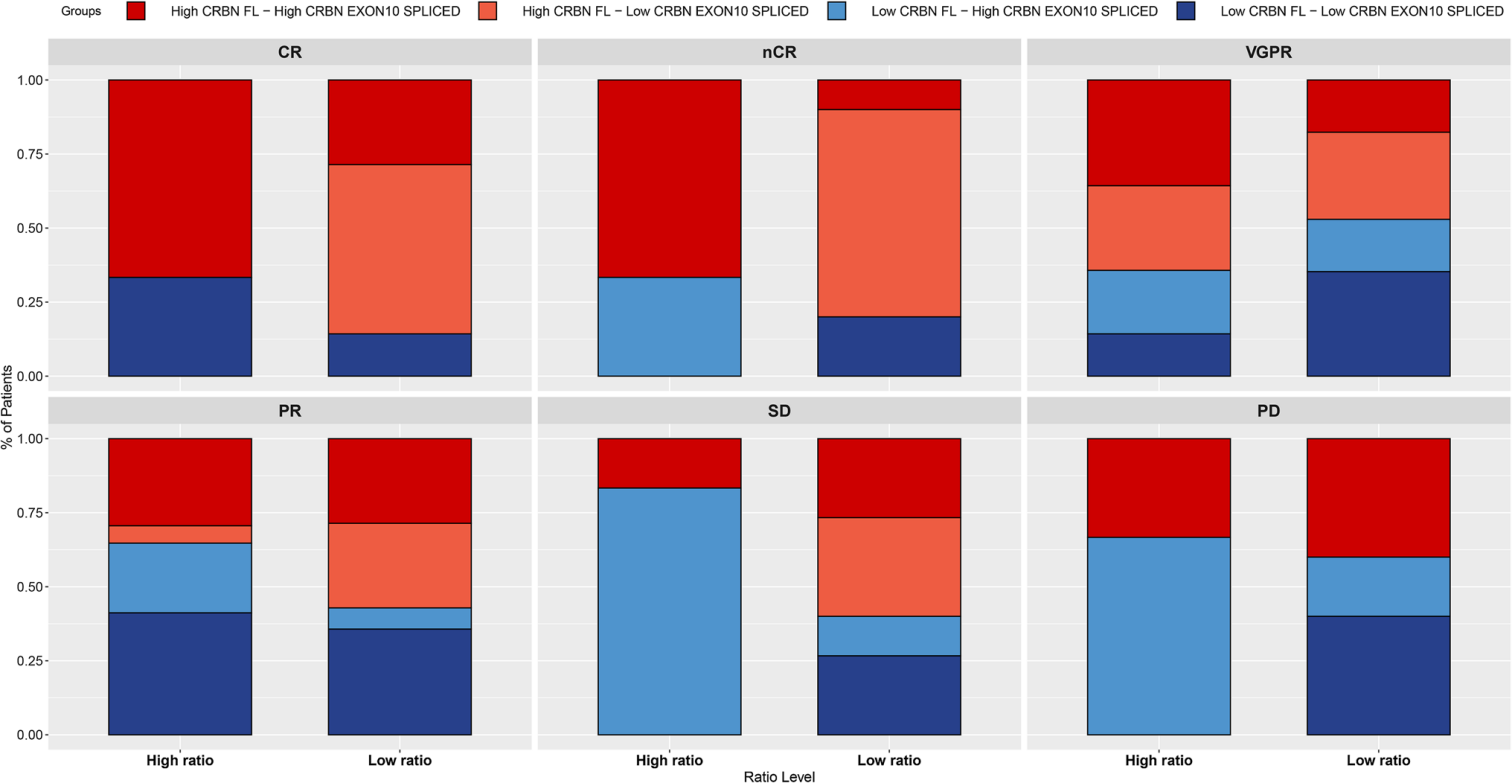
Correlation between *CRBN*-FL and exon10-spliced transcript levels respect to ratio level, and response to IMiDs first-line induction therapy. Histograms show the distribution of both *CRBN* transcript variants, defined as low- and high-level, low- and high-ratio level and response to induction therapy. Groups: High *CRBN*-FL-high *CRBN* exon10-spliced-dark red, high *CRBN*-FL-low *CRBN* exon10-spliced-light red, low *CRBN*-FL-high *CRBN* exon10-spliced-dark blue, low *CRBN*-FL-low *CRBN* exon10-spliced-light blue. CR = complete response, *n*CR = near complete response, VGPR = very good partial response, PD = partial disease, PR = progression disease, SD = stable disease

Considering that the ratio of *CRBN* exon10-spliced (ENST00000424814.5)/*CRBN*-FL (ENST00000231948.8) has previously been proposed to correlate with outcome after IMiD-based regimens [18], we evaluated the response to induction therapy of these patients based on the ratio of transcript levels of spliced *CRBN* to that of *CRBN*-FL. We used the median value of the ratio calculated on all 137 MM samples to determine the optimum cut-off to distinguish patients with high *vs* low ratio. As shown in Fig. 2B, using a cut-off ratio of 0.0595, we found that the ratio was significantly lower in R patients as compared to NR patients (Wilcoxon test, *p* = 0.0084), suggesting a tendency drives by *CRBN*-FL isoform-carrying patients to respond to IMiDs-based induction therapy. To verified this hypothesis, we performed a subgroup analysis by stratifying R and NR patients in different categories based both on their specific response to treatment (i.e., CR, *n*CR, VGPR and SD, PR, and PD, respectively), and the *CRBN* ratio level (i.e., high *vs* low ratio). We observed an increasing number of patients with a high *CRBN* ratio as the response to therapy become suboptimal (data not shown, *p* = 0.0097), confirming the need of a high amount of *CRBN*-FL isoform to achieve an optimal response. Subsequently, to further validate this observation, we assessed whether the distribution of *CRBN* transcripts, both spliced and full-length, defined as high or low level (i.e., high *CRBN*-FL and/or *CRBN* exon10-spliced and low *CRBN*-FL and/or *CRBN* exon10-spliced), was different between R *vs* NR. As shown in Fig. 3, most of the patients achieving at least a VGPR or better response have a higher percentage of *CRBN*-FL isoform compared to those achieving a suboptimal response, regardless of *CRBN* ratio level. Indeed, the expression level of the spliced isoform tends to be higher in NR patients, confirming that the higher presence of *CRBN* exon10-spliced transcript predicts for lack of IMiDs response.

Unfortunately, we observed that patients treated with PIs-IMID are mainly NR (81%, Fisher’s exact test 95% CI 1.041–14.82, *p* = 0.031), which prevents analysis in a homogeneous clinical context. However, no particular distribution of isoforms is observed between the two patient groups distinguished by treatment. Furthermore, in a multivariate model with treatment and ratio, the independent effect of the therapy was not confirmed (*p* = 0.124).

### Correlation between *CRBN* isoforms expression and clinical outcomes

**Fig. 4:**
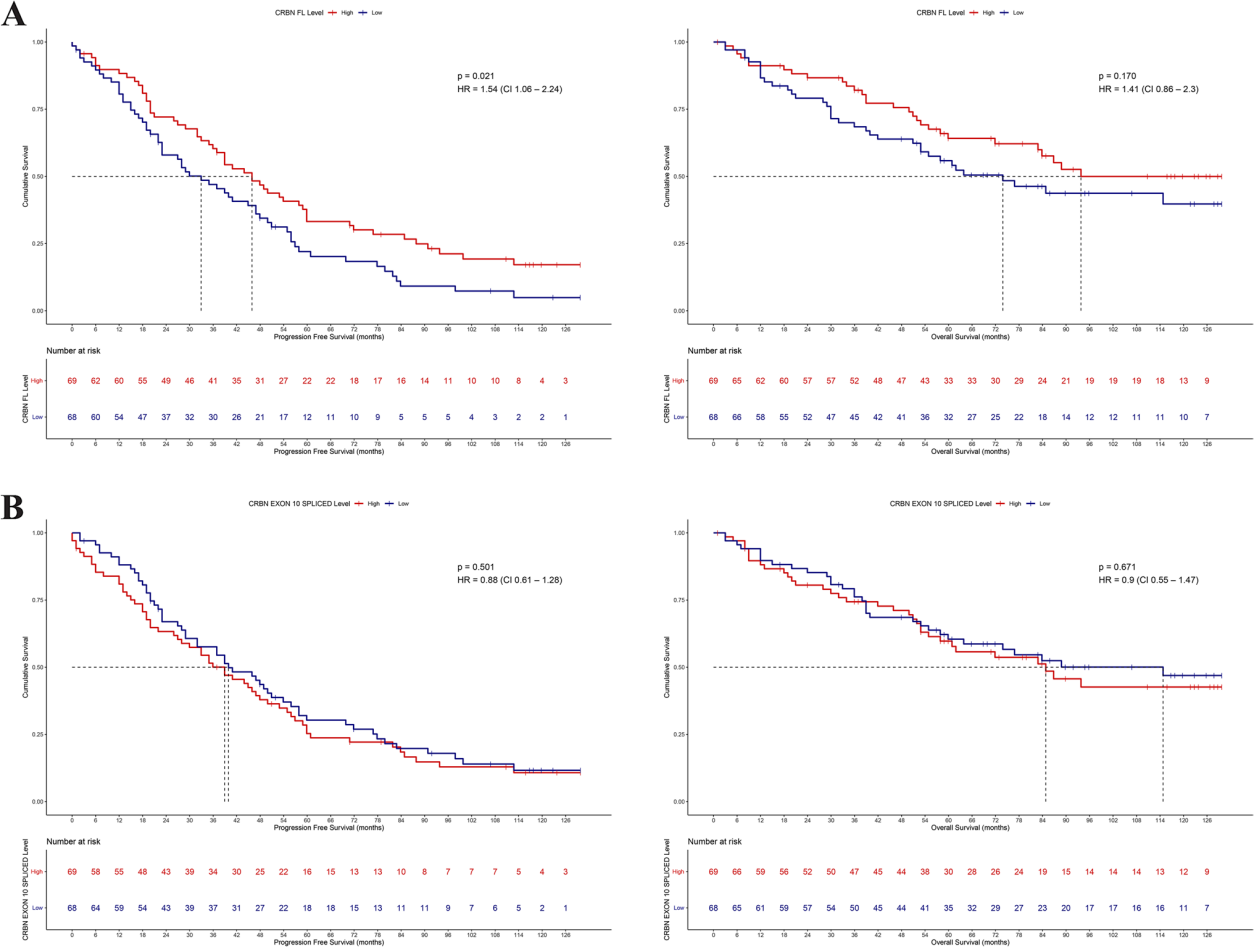
Correlation between *CRBN* isoforms expression and clinical outcome to IMiDs induction therapy in ND myeloma. **A** PFS and OS of non-responder versus responder patients. (PFS: HR 0.4999 [95% CI 0.3371–0.7415] *p* = 0.00042, OS: HR 0.6713 [95% CI 0.4027–1.119] *p* = 0.12). **B** PFS and OS of high *CRBN*-FL versus low *CRBN*-FL expressing patients. (PFS: HR 1.543 [95% CI 1.063–2.24] *p* = 0.021, OS: HR 1.4084 [95% CI 0.8631–2.298] *p* = 0.17). **C** PFS and OS of high *CRBN* exon10-spliced versus low *CRBN* exon10-spliced expressing patients. (PFS: HR 0.8817 [95% CI 0.6096–1.275] *p* = 0.5, OS: HR 0.9012 [95% CI 0.554–1.466] *p* = 0.67)

**Fig. 5:**
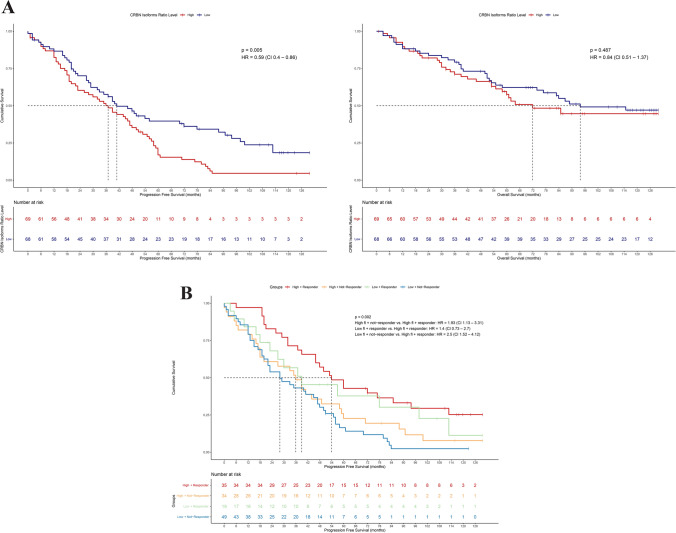
Correlation between *CRBN* ratio level and clinical outcome to IMiDs induction therapy in ND myeloma. **A** PFS and OS of high *CRBN* ratio *vs* low *CRBN* ratio. (PFS: HR 0.588 [95% CI 0.4024–0.8594] *p* = 0.0055, OS: HR 0.8407 [95% CI 0.5142–1.375] *p* = 0.49). **B** PFS curve of the patient groups determined by iteration between *CRBN*-FL levels and clinical response (*p* = 0.002). The risk of each group was calculated compared to the high *CRBN*-FL-R patient group (High *CRBN*-FL + NR, HR: 1.93 [95% CI = 1.13–3.31], *p* = 0.016109; Low *CRBN*-FL + R, HR: 1.4 [95% CI = 0.73–2.7], *p* = 0.309500; Low *CRBN*-FL + NR, HR: 2.5 [95% CI 1.52–4.12] *p* = 0.000324)

We examined the association between *CRBN*’s transcript levels and patients’ outcome. Firstly, we analyzed the clinical outcomes of patients based on *CRBN*-FL isoform levels, and we observed that progression free survival (PFS) was significantly longer in patients expressing high *CRBN*-FL (PFS: HR 0.6481 [95% CI 1.063–2.24], *p* = 0.021) but not in patients with high *CRBN* exon10-spliced variant (PFS: HR 1.1341 [95% CI 0.6096–1.275], *p* = 0.5), confirming the strong association of *CRBN*-FL isoform with higher response to therapy. Indeed, the baseline deleted *CRBN* expression level does not affect patient’s outcome, since both the PFS and OS of patients expressing higher levels of *CRBN* exon10-spliced isoform were not significantly different from those of patients expressing low levels (PFS: HR 0.887 [95% CI 0.6096–1.275], *p* = 0.5; OS: HR 0.9012 [95% CI 0.554–1.466], *p* = 0.675) (Fig. 4A and B). Remarkably, when patients were stratified according to *CRBN* ratio level in high *vs* low ratio, the presence of a high-ratio level was significantly related with worse patients’ outcome, whereas patients expressing a low ratio have a better outcome (PFS: HR 0.588 [95% CI 0.4024–0.8594], *p* = 0.0055; OS: HR 0.8407 [95% CI 0.5142–1.375], *p* = 0,49) (Fig. 5A). In a multivariate model, we evaluated the independent effects of *CRBN*-FL isoform levels and response to therapy on PFS, showing that response has a significant impact on relapse (NR: HR 1.86 [95% CI 0.36–0.81], *p* = 0.00262; *CRBN*-FL: HR 1.3297 [95% CI 0.91–1.95], *p* = 0.14635). However, it was observed that the combination of these variables allows further stratification of NR patients, as those with low *CRBN*-FL level and no response to therapy have a higher risk of relapse (HR 2.5 [95% CI 1.52–4.12] *p* = 0.000324, Fig. 5B), while NR patients with high *CRBN*-FL level have a lower risk (HR 1.93 [95% CI 1.13–3.31], *p* = 0.016109, Fig. 5B).

Additionally, to explore the prognostic impact of *CRBN*-FL levels, we performed a logistic regression model with those factors known to have an impact on response in MM patients and were available for at least 50% of patients. After assessing the correlation of the variables with the response in univariate, the only significant variable was first-line transplantation. As expected, in the multivariate analysis, the probability of first-line response is mainly determined by the use of transplantation in the treatment regimen (OR 3.62; *p* = 0.00135; CI 1.67943–8.16039), and by the presence of high levels of *CRBN*-FL in CD138 + cells (OR 2.58; *p* = 0.01347; CI 1.22746–5.55781) (Supplementary Table 2).

Taken together, these results demonstrated a positive correlation with outcome and *CRBN*-FL expression level, with low percentage of *CRBN*-FL transcript associated with higher probability to unfavorable response to induction therapy.

### *CRBN*-FL low-expressing patients down-regulated pathways involved in immune response

Based on the results described above, we decided to perform an exploratory analysis by comparing the gene expression profile of patients (CoMMpass dataset, *n* = 50) expressing high levels of *CRBN*-FL to those with a low-expression level, to identify differentially expressed pathways and genes in *CRBN*-FL expressing patients, potentially related to response to therapy and eventually to IMiDs resistance.

The baseline clinical and cytogenetic characteristics of the two subgroups of patients were homogeneous, thus suggesting that only the different expression levels of *CRBN* might account for the differentially expressed gene profile (Table 1).

**Fig. 6:**
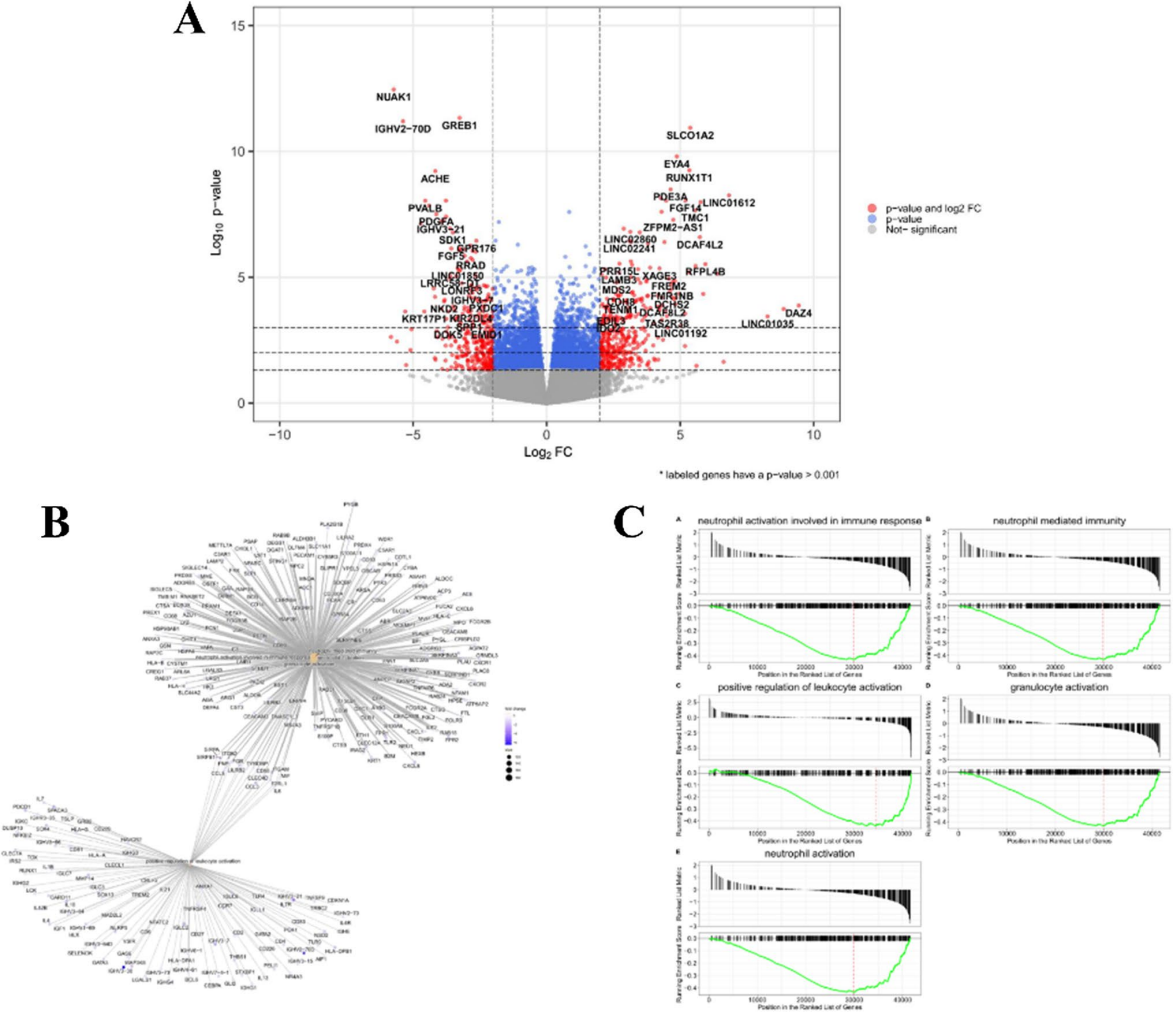
Gene expression and gene set enrichment analysis of low *CRBN*-FL versus high *CRBN*-FL expressing patients. **A** Volcano plot of differential expression gene analysis (DESeq2). Labeled genes have a *p*-value < 0.001. **B** Transcriptional network of the five significantly regulated pathways. **C** Gene set enrichment analysis of the five significantly down-regulated pathways

**Fig. 7:**
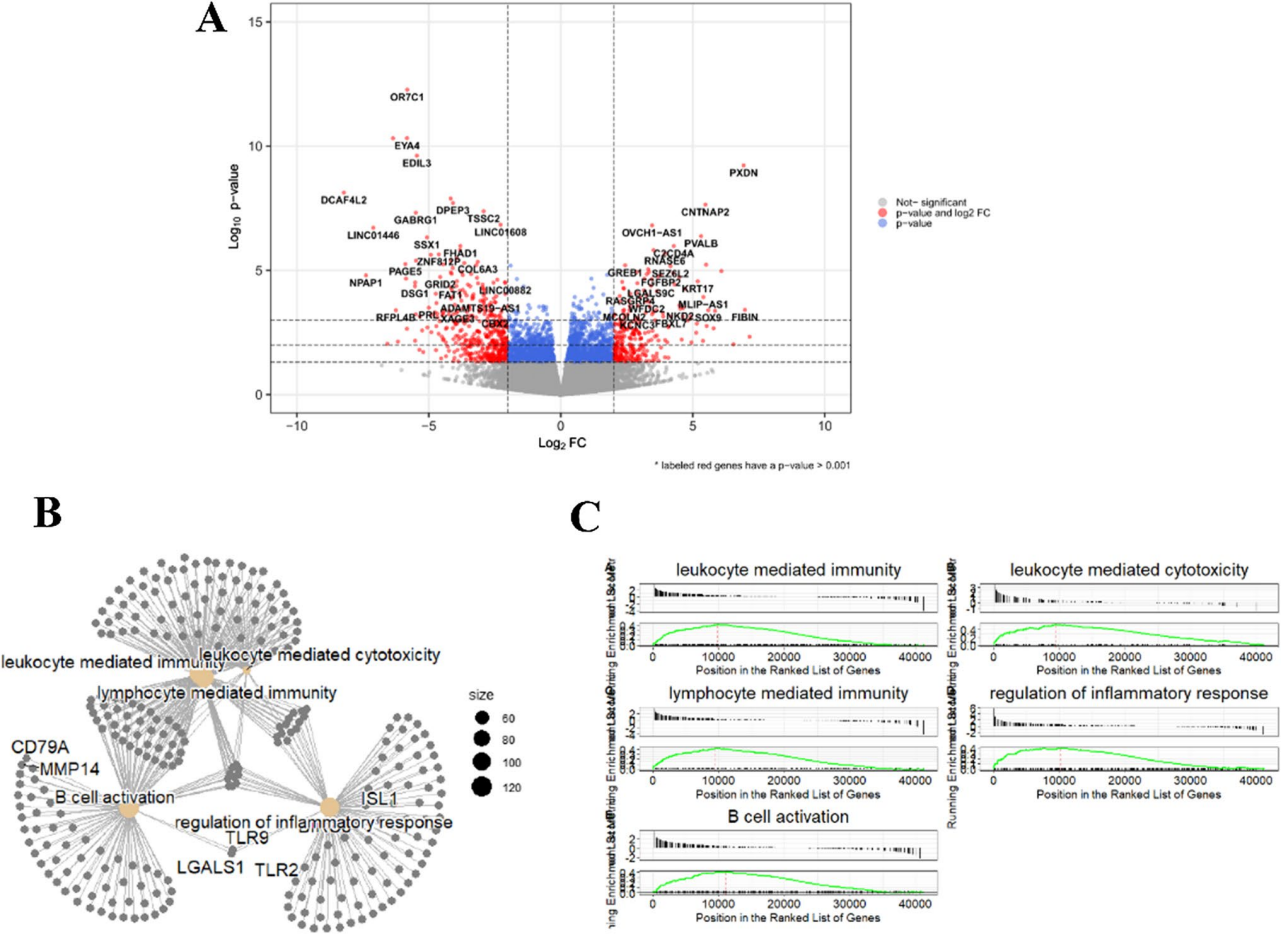
Gene expression and gene set enrichment analysis of low *CRBN*-FL and NR versus high *CRBN*-FL R patients. **A** Volcano plot of differential expression gene analysis (DESeq2). Labeled genes have a *p*-value < 0.001. **B** Transcriptional network of the five significantly regulated pathways. **C** Gene set enrichment analysis of the five significantly up-regulated pathways

Both down- and up-regulated probe-sets were analyzed as described in Material and Methods section, to identify the biological processes, as well as the molecular functions which might be altered in patients expressing low levels of *CRBN*-FL. To this end, to identify genes set significantly deregulated in high *CRBN*-FL-expressing patients as compared to low-expressing ones, the gene-based expression estimation was used to perform a differential expression gene analysis (DEG) between the two subgroups of patients. Overall, 326 probe-sets resulted significantly differentially down-regulated in patients with lower versus those with higher amount of *CRBN*-FL (Fold Change, FC > 2 and *p* ≤ 0.05). As shown in the volcano plot (Fig. 6A) among the down-regulated genes, the most represented were involved in antigen binding activity and immunoglobulin receptor binding activity as well as activation of immune response, accounted mainly for the down-regulation of IGHV2-70D (FC =  − 5.3772, *p* = 2.86e-07), IGHV3-21 (FC =  − 39.556, *p* = 6.20e-10), and IGHV3 (FC =  − 2.78443, *p* = 4.19e-9). Moreover, by gene set enrichment analysis (GSEA), a significant down-modulation of several pathways was observed, including multiple immune system-related signaling pathways, such as neutrophil activation involved in immune response (Enrichment Score, ES =  − 0.43007, *p* = 1e-10), neutrophil-mediated immunity (ES =  − 0.43447, *p* = 1e-10), positive regulation of leukocyte activation (ES =  − 0.44334, *p* = 1e-10), granulocyte activation (ES =  − 0.43266, *p* = 1e-10), and neutrophil activation (ES =  − 0.43505, *p* = 1e-10) which were the most represented (Fig. 6B and C). Overall, these data suggest that plasma cells expressing low levels of *CRBN*-FL might have impaired the competence to correctly interact with other immune cells in the marrow, thus eventually implying a breakage of the MM cells and microenvironment cross-talk in these patients. To confirm this observation, we next compared the transcriptomic profile of low *CRBN*-FL NR patients with high *CRBN*-FL R patients, and we found the up-regulation of several genes involved in positive regulation of acute inflammatory response, (C2CD4A, FC = 4.2823, *p* = 0.00148) and involved in cytotoxic lymphocyte-mediated immunity (FGFBP2, FC = 37.915*, p* = 0.00858) (Fig. 7A). Notably, we observed that among the up-regulated pathways, predominantly, the immune system-related signaling pathways were significantly enriched in *CRBN*-FL R patients (*e.g., leukocyte-mediated immunity* ES = 0.41539, *p* = 4.89e-8, *leukocyte-mediated cytotoxicity* ES = 0.52510, *p* = 1.55e-6, *lymphocyte-mediated immunity* ES = 0.41791, *p* = 1.27e-6, *B cell activation* ES = 0.40423, *p* = 8.92e-6 and *regulation of inflammatory response* ES = 0.40030, *p* = 3.17e-6) (Fig. 7B and C).

Overall, these data strongly support the connection between CRBN and immune system pathway suggesting a potential link with IMiD resistance.

## Discussion

CRBN is the direct binding target of the IMiDs, one of the main classes of drugs in use in different MM phases. There is an increasing amount of evidence indicating that IMiD resistance arises, at least in part, from the acquisition and selection of genetic changes carried by CRBN. In this contest, the accurate quantification of *CRBN* mRNA level represents an issue, since *CRBN* transcript might undergo a complex sequence of alternative splicing, which results in the translation of several isoforms, frequently co-expressed in the same individual. This should be considered, when performing the analysis of the *CRBN* expression, since diverse isoforms have been described, which lack one or more of the CRBN protein functional domains. For instance, the CRBN binding domain of IMiDs, located in the C-terminal region (i.e., Thalidomide-Binding Domain, TBD), [2] is encoded by the *CRBN* exon 10, a region that might be alternatively spliced in MM patients [33, 34].

In this study, we demonstrated that CRBN transcript levels, both full-length and spliced isoforms, correlate with IMiDs response already at diagnosis, and this observation was in line with previously published data [19, 35]. The potential deletion of the drug-binding pocket may lead to a direct loss of drug-binding function of CRBN. However, it is unclear if the relative amount of *CRBN*-FL isoform present in the MM cells may be sufficient to confer drug sensitivity, and/or whether the presence of a high *CRBN* exon10-spliced variant in untreated patients may have a “dominant negative” impact. Indeed, results have been frequently conflicting, and so far, no standard and consistent assays have been set-up, to assess the exact *CRBN* expression level by quantitative PCR, principally for diagnostic purposes. Moreover, these methods actually allow for the quantification of *CRBN*-FL, but do not consider the possible presence of different isoforms [33, 36]. To the best of our knowledge, this study is the first attempt to quantify the absolute expression levels of both *CRBN*-FL and the *CRBN* exon10-spliced variant by analyzing the transcript data of diagnostic samples from NDMM patients who received an IMiDs-based induction therapy. We found that the level of *CRBN* exon10-spliced isoform was associated with inferior IMiD-based treatment response. Notably, patients with high *CRBN*-FL were more likely to improve depth of response compared to those with low level. Additionally, when comparing the ratio between *CRBN* exon10-spliced/*CRBN*-FL transcripts, we demonstrated that this ratio was inversely correlated with response to therapy. Remarkably, we observed that the ratio was significantly lower in R patients rather than NR patients, suggesting the need of high amount of *CRBN*-FL isoform to respond to IMiDs-based induction therapy. This speculation is further endorsed by the finding that patients achieving at least a VGPR or better response have a higher percentage of *CRBN*-FL isoform compared to those achieving a suboptimal response, regardless of *CRBN* ratio level. Moreover, both high *CRBN*-FL-expressing patients and low ratio patients were sufficiently represented at diagnosis, to enabling us to assess correlation, and they were associated with significantly improved PFS. These results strongly support the notion that the accurate expression of *CRBN* could potentially predict the clinical course of MM patients and identify, upfront, patients with a high probability of responding to IMiD-based regimens.

Newer derivatives of IMiDs, CELMoDs, bind target protein CRBN with greater affinity, leading to faster substrate degradation and therefore more potent anti-proliferative effects. Recent data suggest CELMoDs iberdomide (CC-220) and mezigdomide (CC92480) may have efficacy in lenalidomide- and pomalidomide-resistant patients in ongoing clinical trials [37–39]. The association of CELMoD compounds to the TBD has been shown to be both necessary and sufficient to trigger CRBN allosteric rearrangements, leading to the “closure” of the non-canonical “open” conformation [40]. Indeed, CELMoD agents bind to a conserved hydrophobic pocket in CRBN to create a molecular surface, able to recruit downstream target proteins [40, 41]. Importantly, just one of the most critical direct downstream CRBN’s targets (i.e., Ikaros) stably associates with the closed CRBN conformation, highlighting the importance of allostery for CELMoD compound efficacy. In this regard, the allosteric perturbations possibly introduced by mutations and/or aberrations of CRBN gene (i.e., presence of high amount of spliced isoform) should be considered as possible mechanism of drug resistance, in order to improve CELMoD’s clinical activity [40, 41].

Notably, the gene expression profile of *CRBN*-FL-carrying patients showed that among up-regulated pathways, the multiple immune system-related signaling pathways were significantly enriched in patients expressing high levels of *CRBN*-FL variant. These results highlighted an unexpected correlation between *CRBN*-FL level and immune pathway genes within MM cells of untreated patients. However, we cannot exclude that other factor, including transcriptional and/or post-transcriptional mechanisms, CRBN mutations and/or mutations in genes involved in CRBN’s pathways (i.e., in IKZF1 or IKZF3), reflecting the presence of sub-clonal mutations in genes encoding the splicing machinery [19, 21], might be also present and implicated in the acquisition of immunomodulatory drug resistance and subsequent clinical relapse; these additional factors should be further explored.

Moreover, we also analyzed the clinical outcomes of patients based on response to induction therapy (i.e., R *vs* NR), and, as expected, we observed a significant longer PFS in R subgroup compared to NR (PFS: HR 2 [95% CI 0.3371–0.7415]) *p* = 0.00042; conversely, we did not see a significant impact on overall survival (OS) (OS: HR 1.49 [95% CI 0.4027–1.119], *p* = 0.12) (data not shown). Finally, we showed a clear positive association between *CRBN*-FL expression and clinical outcome, highlighting the expression of high levels of *CRBN* exon10-spliced variant in NR patients, even though we did not explore the underlying mechanisms that cause such CRBN aberrations and result in drug resistance. The identification of a clear biological connection between the occurrence of CRBN aberrations and drug resistance was outside the aim of this investigation, and will be the subject of future analyses. Moreover, we acknowledge that the cut-off employed to identify patients with high *CRBN* exon10-spliced isoform might be more precisely defined on larger cohorts of patients. We also recognize that one of the pitfalls of this investigation is that patients included in this study have been treated with regimens that are no longer considered as up-to date, and that further analyses employing patients’ samples who will receive new drugs combinations therapy might strengthen the data.

## Conclusions

Our results highlight the importance to define the amount of deleted *CRBN* isoforms, in order to employ CRBN as a biomarker of IMiDs response already at the diagnosis. To this end, it becomes clear that the precise quantification of *CRBN* variants levels is necessary to correctly determine both the “penetrance” and/or the optimal cut-off aim at defining upfront patients who will lack an IMiD response. What is the right balance between the two *CRBN* isoforms inside tumor cell needed for achieving a good response? This is still an open question that must be answered to have a complete overview and understanding of CRBN-related IMiD resistance mechanisms. In this scenario, it becomes clear that our data must be confirmed employing a large cohort of patients, to introduce a reliable approach in the routine clinical setting; in fact, the importance of a predictive biomarker in a complex genomic disease as MM is crucial, since it may lead to the establishment of personalized-treatment therapy.

### Supplementary Information

Below is the link to the electronic supplementary material.

Supplementary file1 (DOCX 30 kb)

Supplementary file2 (DOCX 13 kb)

## Data Availability

Bioinformatics datasets presented in this study can be found in online repositories, and the datasets used and/or analyzed during experiments are available from the corresponding author on reasonable request.
