# Supplementary material for: High levels of CRBN isoform lacking IMiDs binding domain predicts for a worse response to IMiDs-based upfront therapy in newly diagnosed myeloma patients
Source: Clin Exp Med. 2023 Oct 10;23(8):5227–39. doi: 10.1007/s10238-023-01205-y (PMC10725394; doi:10.1007/s10238-023-01205-y)
Supplement: Supplementary file 1 — Supplementary file1 (DOCX 30 kb) [file 10238_2023_1205_MOESM1_ESM.docx]

**Supplementary Table 1. CRBN isoforms variant expression level and response to therapy.** The CRBN transcript level, both full-length and spliced variants, derived from qPCR and RNAseq experiments (MM-BO and CoMMpass, respectively). R= responder, NR=not responder.

| **N** | **Public ID** | ***CRBN*-FL** | ***CRBN* exon10-spliced** | **Ratio Level** | ***CRBN*-FL category** | ***CRBN* exon10-spliced category** | **Ratio level category** | **First Line Therapy** | **Patients’**  **category** | **Dataset** |
| --- | --- | --- | --- | --- | --- | --- | --- | --- | --- | --- |
| **1** | MPC_917 | 0,048766424 | 0,000562675 | 0,011538163 | High | High | Low ratio | IMiDs | R | MM-BO |
| **2** | MPC_1137 | 0,010253 | 0,000404 | 0,039403102 | Low | Low | Low ratio | IMiDs | R | MM-BO |
| **3** | MPC_2108 | 0,028735356 | 0,000498615 | 0,017351983 | High | High | Low ratio | IMiDs | NR | MM-BO |
| **4** | MPC_1149 | 0,022378439 | 0,000117236 | 0,005238807 | High | Low | Low ratio | IMiDs | NR | MM-BO |
| **5** | MPC_2820 | 0,041998178 | 0,000497705 | 0,011850643 | High | High | Low ratio | IMiDs | NR | MM-BO |
| **6** | MPC_2683 | 0,028673166 | 0,000297634 | 0,010380225 | High | Low | Low ratio | IMiDs | NR | MM-BO |
| **7** | MPC_2279 | 0,022422793 | 0,000609225 | 0,027169888 | High | High | Low ratio | IMiDs | NR | MM-BO |
| **8** | MPC_1556 | 0,03515 | 0,002724 | 0,077496444 | High | High | High ratio | IMiDs | NR | MM-BO |
| **9** | MPC_189 | 0,08716759 | 0,000470104 | 0,005393107 | High | Low | Low ratio | IMiDs | R | MM-BO |
| **10** | MPC_2581 | 0,019723 | 0,001323 | 0,067079045 | Low | High | High ratio | IMiDs | NR | MM-BO |
| **11** | MPC_1981 | 0,024414793 | 0,002280069 | 0,093388853 | High | High | High ratio | IMiDs | NR | MM-BO |
| **12** | MPC_966 | 0,020513 | 0,001269 | 0,061863209 | Low | High | High ratio | IMiDs | R | MM-BO |
| **13** | MPC_3042 | 0,06745137 | 0,000160378 | 0,002377685 | High | Low | Low ratio | IMiDs | R | MM-BO |
| **14** | MPC_1042 | 0,040938065 | 0,000160506 | 0,003920692 | High | Low | Low ratio | IMiDs | R | MM-BO |
| **15** | MPC_2470 | 0,020838 | 0,000669 | 0,032104809 | Low | High | Low ratio | IMiDs | R | MM-BO |
| **16** | MPC_2567 | 0,005973705 | 0,000191227 | 0,032011385 | Low | Low | Low ratio | IMiDs | NR | MM-BO |
| **17** | MPC_1463 | 0,031421347 | 0,000187535 | 0,005968383 | High | Low | Low ratio | IMiDs | R | MM-BO |
| **18** | MPC_1900 | 0,080403356 | 0,002787517 | 0,034669162 | High | High | Low ratio | IMiDs | R | MM-BO |
| **19** | MPC_3010 | 0,012349245 | 0,004187788 | 0,33911287 | Low | High | High ratio | Combined PIs-IMiDs | NR | MM-BO |
| **20** | MPC_2309 | 0,01841145 | 0,000817734 | 0,044414434 | Low | High | Low ratio | IMiDs | NR | MM-BO |
| **21** | MPC_1006 | 0,048742914 | 0,0000426 | 0,000874094 | High | Low | Low ratio | IMiDs | R | MM-BO |
| **22** | MPC_1494 | 0,050265832 | 0,000454516 | 0,009042237 | High | Low | Low ratio | IMiDs | R | MM-BO |
| **23** | MPC_2453 | 0,002263578 | 0,000285612 | 0,126177433 | Low | Low | High ratio | IMiDs | R | MM-BO |
| **24** | MPC_2697 | 0,051272372 | 0,000167314 | 0,003263247 | High | Low | Low ratio | IMiDs | R | MM-BO |
| **25** | MPC_2718 | 0,022207863 | 0,002517065 | 0,113341148 | High | High | High ratio | IMiDs | R | MM-BO |
| **26** | MPC_2788 | 0,161900937 | 0,0000453 | 0,000279916 | High | Low | Low ratio | IMiDs | R | MM-BO |
| **27** | MPC_2930 | 0,035276 | 0,001286 | 0,03645538 | High | High | Low ratio | IMiDs | R | MM-BO |
| **28** | MPC_2975 | 0,023603148 | 0,000633051 | 0,026820616 | High | High | Low ratio | IMiDs | R | MM-BO |
| **29** | MPC_1159 | 0,006561839 | 0,0000683 | 0,010402486 | Low | Low | Low ratio | IMiDs | R | MM-BO |
| **30** | MPC_1683 | 0,022179523 | 0,00399639 | 0,180183762 | High | High | High ratio | IMiDs | R | MM-BO |
| **31** | MPC_1806 | 0,031197422 | 0,0000494 | 0,001583592 | High | Low | Low ratio | IMiDs | R | MM-BO |
| **32** | MPC_2041 | 0,100466407 | 0,000187513 | 0,001866425 | High | Low | Low ratio | IMiDs | R | MM-BO |
| **33** | MPC_2512 | 0,053953986 | 0,0000546 | 0,001012145 | High | Low | Low ratio | IMiDs | R | MM-BO |
| **34** | MPC_2568 | 0,011121 | 0,001872 | 0,168330186 | Low | High | High ratio | IMiDs | R | MM-BO |
| **35** | MPC_2781 | 0,032806 | 0,002396 | 0,07303542 | High | High | High ratio | IMiDs | R | MM-BO |
| **36** | MPC_2875 | 0,017869 | 0,000223 | 0,012479713 | Low | Low | Low ratio | IMiDs | R | MM-BO |
| **37** | MPC_1002 | 0,005744682 | 0,000140726 | 0,024496766 | Low | Low | Low ratio | IMiDs | NR | MM-BO |
| **38** | MPC_1048 | 0,013630877 | 0,000493609 | 0,036212569 | Low | High | Low ratio | IMiDs | NR | MM-BO |
| **39** | MPC_1234 | 0,041946675 | 0,001689283 | 0,040272159 | High | High | Low ratio | IMiDs | NR | MM-BO |
| **40** | MPC_1436 | 0,003695489 | 0,000521605 | 0,141146477 | Low | High | High ratio | IMiDs | NR | MM-BO |
| **41** | MPC_1462 | 0,018354016 | 0,001881424 | 0,102507473 | Low | High | High ratio | IMiDs | NR | MM-BO |
| **42** | MPC_1470 | 0,047693814 | 0,0000260 | 0,000545394 | High | Low | Low ratio | IMiDs | NR | MM-BO |
| **43** | MPC_1897 | 0,075939055 | 0,000522965 | 0,006886643 | High | High | Low ratio | IMiDs | NR | MM-BO |
| **44** | MPC_2089 | 0,003337 | 0,000182 | 0,054540006 | Low | Low | Low ratio | IMiDs | NR | MM-BO |
| **45** | MPC_2130 | 0,02397861 | 0,001213904 | 0,050624442 | High | High | Low ratio | IMiDs | NR | MM-BO |
| **46** | MPC_2189 | 0,014973661 | 0,000891138 | 0,059513716 | Low | High | High ratio | IMiDs | NR | MM-BO |
| **47** | MPC_2280 | 0,184509039 | 0,000275804 | 0,001494798 | High | Low | Low ratio | IMiDs | NR | MM-BO |
| **48** | MPC_2291 | 0,015885 | 0,000995 | 0,062637709 | Low | High | High ratio | IMiDs | NR | MM-BO |
| **49** | MPC_2296 | 0,020269 | 0,003593 | 0,177265775 | Low | High | High ratio | IMiDs | NR | MM-BO |
| **50** | MPC_2314 | 0,015896985 | 0,0000773 | 0,004864676 | Low | Low | Low ratio | IMiDs | NR | MM-BO |
| **51** | MPC_2333 | 0,009638451 | 0,000113263 | 0,011751189 | Low | Low | Low ratio | IMiDs | NR | MM-BO |
| **52** | MPC_2638 | 0,010786216 | 0,000607573 | 0,056328649 | Low | High | Low ratio | IMiDs | NR | MM-BO |
| **53** | MPC_265 | 0,065805 | 0,000365 | 0,005546691 | High | Low | Low ratio | IMiDs | NR | MM-BO |
| **54** | MPC_2863 | 0,065079437 | 0,004548937 | 0,06989822 | High | High | High ratio | IMiDs | NR | MM-BO |
| **55** | MPC_2955 | 0,013382355 | 0,002073818 | 0,154966577 | Low | High | High ratio | IMiDs | NR | MM-BO |
| **56** | MPC_2982 | 0,018861463 | 0,002498886 | 0,132486303 | Low | High | High ratio | IMiDs | NR | MM-BO |
| **57** | MPC_3050 | 0,006851971 | 0,000597285 | 0,087169802 | Low | High | High ratio | IMiDs | NR | MM-BO |
| **58** | MPC_996 | 0,025465968 | 0,000446031 | 0,017514777 | High | Low | Low ratio | IMiDs | NR | MM-BO |
| **59** | MPC_1337 | 0,000982664 | 0,000856323 | 0,871430787 | Low | High | High ratio | IMiDs | NR | MM-BO |
| **60** | MPC_1039 | 0,030516473 | 0,000643197 | 0,021077054 | High | High | Low ratio | IMiDs | NR | MM-BO |
| **61** | MPC_1410 | 0,075239018 | 0,001064594 | 0,014149498 | High | High | Low ratio | IMiDs | NR | MM-BO |
| **62** | MPC_1588 | 0,019337976 | 0,0016185 | 0,0836954 | Low | High | High ratio | IMiDs | NR | MM-BO |
| **63** | MPC_1816 | 0,035298854 | 0,000719326 | 0,02037816 | High | High | Low ratio | IMiDs | NR | MM-BO |
| **64** | MPC_2302 | 0,021798985 | 0,000397263 | 0,018223917 | High | Low | Low ratio | IMiDs | NR | MM-BO |
| **65** | MPC_2714 | 0,017623426 | 0,000281432 | 0,015969223 | Low | Low | Low ratio | IMiDs | NR | MM-BO |
| **66** | MPC_3058 | 0,047943201 | 0,000206005 | 0,004296857 | High | Low | Low ratio | IMiDs | NR | MM-BO |
| **67** | MPC_3153 | 0,008686553 | 0,000157846 | 0,018171256 | Low | Low | Low ratio | IMiDs | NR | MM-BO |
| **68** | MPC_864 | 0,017618814 | 0,0000850 | 0,004822096 | Low | Low | Low ratio | IMiDs | NR | MM-BO |
| **69** | MPC_1348 | 0,008026403 | 0,000526578 | 0,065605749 | Low | High | High ratio | IMiDs | NR | MM-BO |
| **70** | MPC_1823 | 0,013135813 | 0,00012416 | 0,009452004 | Low | Low | Low ratio | IMiDs | NR | MM-BO |
| **71** | MPC_1862 | 0,004684603 | 0,000223791 | 0,047771594 | Low | Low | Low ratio | IMiDs | NR | MM-BO |
| **72** | MPC_2463 | 0,034857061 | 0,000826064 | 0,023698603 | High | High | Low ratio | IMiDs | NR | MM-BO |
| **73** | MPC_3084 | 0,012842992 | 0,00071892 | 0,055977633 | Low | High | Low ratio | IMiDs | NR | MM-BO |
| **74** | MPC_1028 | 0,033065 | 0,000419 | 0,01267201 | High | Low | Low ratio | IMiDs | R | MM-BO |
| **75** | MPC_1291 | 0,018239682 | 0,0000828 | 0,004537989 | Low | Low | Low ratio | IMiDs | R | MM-BO |
| **76** | MPC_137 | 0,015952936 | 0,000386087 | 0,02420164 | Low | Low | Low ratio | IMiDs | R | MM-BO |
| **77** | MPC_1379 | 0,009927441 | 0,0000835 | 0,008409945 | Low | Low | Low ratio | IMiDs | R | MM-BO |
| **78** | MPC_179 | 0,008123903 | 0,0000419 | 0,005153778 | Low | Low | Low ratio | IMiDs | R | MM-BO |
| **79** | MPC_1966 | 0,001344257 | 0,000190074 | 0,141397163 | Low | Low | High ratio | IMiDs | R | MM-BO |
| **80** | MPC_231 | 0,23619918 | 0,001573296 | 0,006660887 | High | High | Low ratio | IMiDs | R | MM-BO |
| **81** | MPC_2699 | 0,014138 | 0,000659 | 0,046611968 | Low | High | Low ratio | IMiDs | R | MM-BO |
| **82** | MPC_2783 | 0,054070415 | 0,000437658 | 0,008094225 | High | Low | Low ratio | IMiDs | R | MM-BO |
| **83** | MPC_2793 | 0,01743 | 0,000795 | 0,045611015 | Low | High | Low ratio | IMiDs | R | MM-BO |
| **84** | MPC_2804 | 0,013061255 | 0,000189565 | 0,014513529 | Low | Low | Low ratio | IMiDs | R | MM-BO |
| **85** | MPC_3166 | 0,021939 | 0,000371 | 0,016910525 | High | Low | Low ratio | IMiDs | R | MM-BO |
| **86** | MPC_81 | 0,006861449 | 0,0000333 | 0,004859821 | Low | Low | Low ratio | IMiDs | R | MM-BO |
| **87** | MPC_855 | 0,04458 | 0,002405 | 0,053947959 | High | High | Low ratio | IMiDs | R | MM-BO |
| **88** | MMRF_1024 | 3,549208 | 0,512824 | 0,1444897 | Low | Low | High ratio | Combined PIs-IMiDs | NR | CoMMpass |
| **89** | MMRF_1033 | 4,695154 | 0,48969 | 0,104296898 | Low | Low | High ratio | IMiDs | NR | CoMMpass |
| **90** | MMRF_1038 | 8,611935 | 0,770362 | 0,089452835 | High | Low | High ratio | IMiDs | NR | CoMMpass |
| **91** | MMRF_1108 | 4,511498 | 0,662928 | 0,146941881 | Low | Low | High ratio | Combined PIs-IMiDs | NR | CoMMpass |
| **92** | MMRF_1129 | 7,472334 | 1,028167 | 0,137596499 | High | High | High ratio | IMiDs | NR | CoMMpass |
| **93** | MMRF_1137 | 6,332963 | 0,568981 | 0,089844359 | High | Low | High ratio | IMiDs | NR | CoMMpass |
| **94** | MMRF_1164 | 1,920039 | 1,535958 | 0,799961876 | Low | High | High ratio | Combined PIs-IMiDs | NR | CoMMpass |
| **95** | MMRF_1169 | 7,761944 | 0,732453 | 0,094364633 | High | Low | High ratio | Combined PIs-IMiDs | NR | CoMMpass |
| **96** | MMRF_1184 | 8,609777 | 1,695772 | 0,196958876 | High | High | High ratio | IMiDs | NR | CoMMpass |
| **97** | MMRF_1202 | 2,273502 | 0,275206 | 0,121049377 | Low | Low | High ratio | Combined PIs-IMiDs | NR | CoMMpass |
| **98** | MMRF_1250 | 4,351202 | 0,639215 | 0,146905384 | Low | Low | High ratio | IMiDs | NR | CoMMpass |
| **99** | MMRF_1300 | 9,95955 | 1,300162 | 0,130544251 | High | High | High ratio | Combined PIs-IMiDs | NR | CoMMpass |
| **100** | MMRF_1309 | 3,775234 | 0,54693 | 0,144873139 | Low | Low | High ratio | Combined PIs-IMiDs | NR | CoMMpass |
| **101** | MMRF_1318 | 3,489932 | 1,124205 | 0,322128053 | Low | High | High ratio | Combined PIs-IMiDs | NR | CoMMpass |
| **102** | MMRF_1325 | 2,492478 | 0,257835 | 0,103445246 | Low | Low | High ratio | Combined PIs-IMiDs | NR | CoMMpass |
| **103** | MMRF_1355 | 3,406078 | 0,320893 | 0,094211877 | Low | Low | High ratio | IMiDs | NR | CoMMpass |
| **104** | MMRF_1358 | 3,538016 | 1,035492 | 0,292675895 | Low | High | High ratio | Combined PIs-IMiDs | NR | CoMMpass |
| **105** | MMRF_1436 | 4,95493 | 0,431401 | 0,087065004 | High | Low | High ratio | IMiDs | NR | CoMMpass |
| **106** | MMRF_1470 | 8,210157 | 0,545784 | 0,066476682 | High | Low | High ratio | IMiDs | R | CoMMpass |
| **107** | MMRF_1491 | 1,012467 | 3,790971 | 3,744290925 | Low | High | High ratio | IMiDs | NR | CoMMpass |
| **108** | MMRF_1497 | 2,237079 | 0,767516 | 0,343088465 | Low | Low | High ratio | Combined PIs-IMiDs | NR | CoMMpass |
| **109** | MMRF_1499 | 15,259089 | 1,294089 | 0,08480775 | High | High | High ratio | IMiDs | R | CoMMpass |
| **110** | MMRF_1516 | 5,116657 | 0,288538 | 0,056391898 | High | Low | Low ratio | IMiDs | NR | CoMMpass |
| **111** | MMRF_1574 | 6,425333 | 0,687951 | 0,107068536 | High | Low | High ratio | IMiDs | R | CoMMpass |
| **112** | MMRF_1579 | 2,059668 | 1,044809 | 0,507270589 | Low | High | High ratio | IMiDs | NR | CoMMpass |
| **113** | MMRF_1614 | 11,193377 | 0,950013 | 0,084872778 | High | High | High ratio | IMiDs | R | CoMMpass |
| **114** | MMRF_1796 | 6,744617 | 0,938911 | 0,139208942 | High | High | High ratio | Combined PIs-IMiDs | NR | CoMMpass |
| **115** | MMRF_1810 | 7,813658 | 1,452548 | 0,185898589 | High | High | High ratio | Combined PIs-IMiDs | NR | CoMMpass |
| **116** | MMRF_1932 | 16,784168 | 3,476838 | 0,207149857 | High | High | High ratio | IMiDs | NR | CoMMpass |
| **117** | MMRF_1951 | 4,898605 | 0,766805 | 0,156535381 | Low | Low | High ratio | IMiDs | NR | CoMMpass |
| **118** | MMRF_1997 | 12,568725 | 1,313492 | 0,104504793 | High | High | High ratio | Combined PIs-IMiDs | NR | CoMMpass |
| **119** | MMRF_2012 | 8,171392 | 1,222052 | 0,149552488 | High | High | High ratio | IMiDs | R | CoMMpass |
| **120** | MMRF_2055 | 1,258245 | 1,184049 | 0,941032152 | Low | High | High ratio | IMiDs | R | CoMMpass |
| **121** | MMRF_2057 | 4,929715 | 0,979252 | 0,198642721 | Low | High | High ratio | IMiDs | NR | CoMMpass |
| **122** | MMRF_2087 | 6,034211 | 1,150265 | 0,190623927 | High | High | High ratio | Combined PIs-IMiDs | R | CoMMpass |
| **123** | MMRF_2098 | 1,218818 | 0,464901 | 0,381435949 | Low | Low | High ratio | IMiDs | NR | CoMMpass |
| **124** | MMRF_2113 | 1,218914 | 0,104776 | 0,085958484 | Low | Low | High ratio | Combined PIs-IMiDs | R | CoMMpass |
| **125** | MMRF_2140 | 3,774031 | 0,141439 | 0,037476905 | Low | Low | Low ratio | Combined PIs-IMiDs | NR | CoMMpass |
| **126** | MMRF_2143 | 4,619517 | 0,77966 | 0,16877522 | Low | High | High ratio | IMiDs | R | CoMMpass |
| **127** | MMRF_2290 | 6,458707 | 0,7915 | 0,122547748 | High | High | High ratio | IMiDs | NR | CoMMpass |
| **128** | MMRF_2302 | 6,507314 | 1,175708 | 0,180674853 | High | High | High ratio | Combined PIs-IMiDs | R | CoMMpass |
| **129** | MMRF_2379 | 2,925885 | 0,829849 | 0,283623246 | Low | High | High ratio | IMiDs | R | CoMMpass |
| **130** | MMRF_2471 | 9,164225 | 0,778069 | 0,08490287 | High | High | High ratio | Combined PIs-IMiDs | NR | CoMMpass |
| **131** | MMRF_2476 | 12,065929 | 0,472779 | 0,039182975 | High | Low | Low ratio | IMiDs | NR | CoMMpass |
| **132** | MMRF_2497 | 4,933951 | 0,523741 | 0,106150426 | Low | Low | High ratio | IMiDs | R | CoMMpass |
| **133** | MMRF_2499 | 2,461314 | 0,19861 | 0,080692671 | Low | Low | High ratio | Combined PIs-IMiDs | NR | CoMMpass |
| **134** | MMRF_2505 | 2,086854 | 0,282388 | 0,135317564 | Low | Low | High ratio | Combined PIs-IMiDs | R | CoMMpass |
| **135** | MMRF_2506 | 13,44529 | 1,005146 | 0,074758224 | High | High | High ratio | IMiDs | NR | CoMMpass |
| **136** | MMRF_2525 | 14,598813 | 2,042276 | 0,139893291 | High | High | High ratio | IMiDs | R | CoMMpass |
| **137** | MMRF_2568 | 10,231358 | 0,411997 | 0,040268066 | High | Low | Low ratio | IMiDs | R | CoMMpass |
