# Supplementary material for: High levels of CRBN isoform lacking IMiDs binding domain predicts for a worse response to IMiDs-based upfront therapy in newly diagnosed myeloma patients
Source: Clin Exp Med. 2023 Oct 10;23(8):5227–39. doi: 10.1007/s10238-023-01205-y (PMC10725394; doi:10.1007/s10238-023-01205-y)
Supplement: Supplementary file 2 — Supplementary file2 (DOCX 13 kb) [file 10238_2023_1205_MOESM2_ESM.docx]

**Supplementary Table 2.** **Logistic regression model.** SE=Standard Error; CI=Confident Interval; ASCT=Autologous Stem Cell Transplantation; *CRBN*-FL=*CRBN* full length.

| Term | Odds-Ratio | SE | Statistic | *p* Value | CI 5% | CI 95% |
| --- | --- | --- | --- | --- | --- | --- |
| (Intercept) | 0,19 | 0,38846 | -4,23 | 0,00 | 0,08579 | 0,39718 |
| ASCT (Y) | 3,62 | 0,40114 | 3,21 | 0,00135 | 1,68 | 8,16 |
| *CRBN*-FL | 2,58 | 0,38376 | 2,47 | 0,01347 | 1,23 | 5,56 |
